# Cross-sectional area of the paraspinal muscles and its association with muscle strength among fighter pilots: a 5-year follow-up

**DOI:** 10.1186/s12891-019-2551-y

**Published:** 2019-04-16

**Authors:** Tuomas Honkanen, Matti Mäntysaari, Tuomo Leino, Janne Avela, Liisa Kerttula, Ville Haapamäki, Heikki Kyröläinen

**Affiliations:** 1 Centre for Military Medicine, P.O.Box 50, FI-00301 Helsinki, Finland; 2 Air Force Command Finland, Tikkakoski, Finland; 3 Neuromuscular Research Center, Faculty of Sport and Health Sciences, University of Jyväskylä, Jyväskylä, Finland; 4 HUS Medical Imaging Center, Helsinki, Finland; 5 Department of Leadership and Military Pedagogy, National Defense University, Helsinki, Finland

**Keywords:** Low back pain, MRI, Muscle composition, Isometric strength, Military aviation, G-force

## Abstract

**Background:**

A small cross sectional area (CSA) of the paraspinal muscles may be related to low back pain among military aviators but previous studies have mainly concentrated on spinal disc degeneration. Therefore, the primary aim of the study was to investigate the changes in muscle CSA and composition of the psoas and paraspinal muscles during a 5-year follow up among Finnish Air Force (FINAF) fighter pilots.

**Methods:**

Study population consisted of 26 volunteered FINAF male fighter pilots (age: 20.6 (±0.6) at the baseline). The magnetic resonance imaging (MRI) examinations were collected at baseline and after 5 years of follow-up. CSA and composition of the paraspinal and psoas muscles were obtained at the levels of 3–4 and 4–5 lumbar spine. Maximal isometric strength tests were only performed on one occasion at baseline.

**Results:**

The follow-up comparisons indicated that the mean CSA of the paraspinal muscles increased (*p* < 0.01) by 8% at L3–4 level and 7% at L4–5 level during the 5-year period. There was no change in muscle composition during the follow-up period. The paraspinal and psoas muscles’ CSA was positively related to overall maximal isometric strength at the baseline. However, there was no association between LBP and muscle composition or CSA.

**Conclusions:**

The paraspinal muscles’ CSA increased among FINAF fighter pilots during the first 5 years of service. This might be explained by physically demanding work and regular physical activity. However, no associations between muscle composition or CSA and low back pain (LBP) experienced were observed after the five-year follow-up.

## Background

Low back pain (LBP) is a common disorder throughout Western society [1] and fighter pilots are no exception to that [2, 3]. The reported LBP prevalence among Finnish Air Force (FINAF) fighter pilots is 71% [4], and it is not uncommon that pilots are limited to fly due to spinal disorders [unpublished observations, 2017]. Fighter pilots report higher prevalence of back pain compared to transport or cargo pilots [4, 5]. Therefore, the high acceleration forces have been suggested as an underlying factor for LBP among fighter pilots [3]. Furthermore, it has been found out that the FINAF fighter pilots, who have passed their fast jet flight training, have already experienced flight-induced musculoskeletal pain in their early flight career [3].

Lumbar paraspinal muscle size, asymmetry and composition assessed with Magnetic Resonance Imagining (MRI) have been associated with LBP [6–8]. The paraspinal muscles are smaller in patients with chronic LBP than in their control counterparts [7, 9]. Furthermore, the cross-sectional area (CSA) of the paraspinal muscles, especially at the lowest level of the lumbar spine, has been found to be smaller in LBP patients compared to their healthy counterparts [10]. It has also been suggested that the side-to-side CSA asymmetries of the lumbar paraspinal muscles associate with LBP [10–12]. According to literature, it is, however, conflicting when asymmetries are diagnosed as an abnormality. Hides et al. [11] suggested that asymmetries of greater than 10% should be regarded as an abnormality, whereas Niemeläinen et al. [13] found that the side-to-side paraspinal muscle asymmetries of greater than 10% is common among men without a history of LBP.

The predictive role of paraspinal muscle CSA, asymmetry and composition on LBP is not clear. Some studies [8] have suggested that greater paraspinal fatty infiltration is associated with a higher risk of having LBP, while other studies [10, 14] have not been able to make the same conclusion. According to Lee et al. [7], CSA of the paraspinal muscles at the lower lumbar level can be considered to be a prognostic factor of chronicity of LBP. However, atrophy of the paraspinal muscles may be a consequence of LBP. It is suggested that disc or nerve root damage could cause selective atrophy of the multifidus muscles [15]. Therefore, it has to be carefully considered whether the reduced muscle CSA predicts LBP or vice versa.

It has been suggested that regular (2–3 times per week) resistance training enhances hypertrophy in the paraspinal and psoas muscles [16]. Respectively, it has been found that the paraspinal and psoas muscle CSA correlates with maximal trunk extension and flexion forces [17] and with isokinetic strength [18]. When CSA of the paraspinal and psoas muscles has been compared between athletes and non-athletes, the athletes have had significantly greater CSA in both muscles [17]. There are also conflicting results of a relationship between paraspinal muscle CSA and strength of the lower back muscles. Ropponen et al. [19] found only low associations between the erector (*r* = 0.21) and psoas (*r* = 0.31) muscles’ CSA and isokinetic force. On the contrary, Parkkola et al. [16] were not able to find an association between the back muscles’ size and maximal isometric extension strength of the trunk.

Despite the high incidence of LBP among fighter pilots and the physically demanding high acceleration environment, no previous studies have assessed lumbar paraspinal muscle composition and CSA among fighter pilots. Furthermore, there are no studies investigating the relationship between the isometric muscular strength and muscle CSA and composition among fighter pilots. Previous research, assessing the relationship between muscle composition or CSA and LBP or muscle strength, have focused on patients with LBP or patients and their matched controls [16] or cohorts drawn from population-based samples of working age people [14, 19, 20]. Some studies have also only concentrated on healthy individuals [13, 21], while only two studies have used subjects under middle age [17, 21]. The changes in the psoas and paraspinal muscles of young adults (age ranging from 20 to 26 years) are not documented in any longitudinal follow-up studies.

The main objective of the present study was to investigate the possible changes in CSA and composition of the psoas and paraspinal muscles in the 5-year follow up among the FINAF fighter pilots during their early flight career and, thus to determine whether muscle CSA and composition could have a predictive role for LBP. In addition, the secondary aim was to examine a possible relationship between the overall isometric strength test results and muscle CSA at the baseline. Prevention of pilots’ LBP induced flight duty limitations has enormous operational and economic importance, in addition to protecting pilots’ health. Early identification of pilots susceptible to severe LBP would allow directing the preventive interventions to the risk group. Measurement of low back mobility and muscular function has not been very successful in predicting LBP in (fighter) pilots. Therefore, new methods are needed for this purpose, like the MRI measurement of lumbar paraspinal muscle composition and CSA used in the present study.

## Methods

### Subjects

Study subjects (*n* = 26) were Finnish Air Force (FINAF) fighter pilot volunteers. Their mean (±SD) age was 20.6 (0.6) years at the baseline. All subjects were male pilots. Subject characteristics including weight, physical test results and LBP history are presented in Table 1.

**Table 1:** Baseline and follow-up characteristics of the subjects (*n* = 26), mean (± SD)

	Baseline	Follow-up
Age (yrs.)	20.6 ± 0.6	25.8 (0.7)
Body mass (kg)^a^	76.8 ± 5.7	78.5 (5.6)*
Leg Extension (kg)	221.0 ± 37.9	N/A
Trunk Flexion (kg)	16.9 ± 3.4	N/A
Trunk Extension (kg)	17.4 ± 3.6	N/A
12-min running test (m)	2999 ± 228	N/A
LBP (no. subjects)	0	8

*LBP* low back pain experienced; ^a^ANOVA for repeated measures, (Wilks’ Lambda) was used to obtain *P values;* *p < 0.05

The magnetic resonance imaging (MRI) examinations were collected as a part of a larger study investigating relationships between the high +Gz acceleration exposure in high performance fighter flying and degenerative changes in intervertebral discs. At the beginning of the study, the baseline MRI was obtained and its follow-up five years later. The strength tests were performed within two months after the baseline MRI as a part of regular fitness testing among fighter pilots. The research was approved by the ethics committee of the Central Finland Health Care District, and written informed consent was obtained from all subjects.

Axial T2-weighted MRI were obtained at the levels of the 3–4 and 4–5 lumbar intervertebral discs using a 1.5 T GE Signa HDxt (Milwaukee, WI, USA) with a phased-array surface coil. CSA of both sides of the paraspinal and psoas muscles were measured with Agfa Impax workstation software (Mortsel, Belgium) by tracing the borders of these muscles and were expressed as cm^2^. Each muscle structure was circumscribed by two well-experienced radiologists (both specialized to musculoskeletal radiology) and the average value was calculated from these measures.

It has been found out that the borders between the multifidus and the erector spinae muscles (iliocostalis lumborum and longissimus thoracis pars lumborum) are often difficult to distinguish [22]. Therefore, the multifidus and erector spinae muscles were measured including the non-muscular tissue between them, together as one muscle mass, and considered as the paraspinal muscles. L3-L4 and L4-L5 were selected for the analysis because both of these levels have been used in previous studies [13, 22] and because CSA of the paraspinal muscles has previously been found to be the largest overall at the L3-L4 level [22].

The reliability of MRI in quantifying the paraspinal muscles has been investigated in several studies and the method has constantly been found to be reliable [23, 24]. The ICCs for intrarater reliability for CSA measurements at the level of the 3–4 and 4–5 lumbar intervertebral discs has been reported to be excellent in the psoas (ICC 0.97–0.99), erector spinae (ICC 0.97–0.99) and multifidus muscles (ICC 0.97–0.98). Outcomes for the left and right side are reported separately because the side-to-side paraspinal muscle asymmetry has been found to be common [13].

In addition to the CSA measures, a qualitative muscle composition measurement was conducted by two well-experienced musculoskeletal radiologists. The atrophy of muscle was rated qualitatively for the paraspinal muscles and psoas muscles at the L3–L4 and L4-L5 levels for all subjects based on visual evaluation using a 3-point visual scale (0 = significant muscle atrophy; 1 = minor deposits of non-muscle tissue (e.g. fat), atrophy 2 = normal muscle, no apparent non-muscle tissue). The average value was calculated from these measures. The MRI measurements of muscle morphology and CSA offer valid assessment of muscularity [24], as compared to muscle function tests that may be influenced by such factors as pain and motivation.

### Muscle strength measures

Prior to all muscle strength tests, the pilots performed a standardized 20 min warm-up. It included light jogging for the first five minutes followed by core and mobility exercises guided by a physiotherapist. The tests were carefully introduced to the subjects and in all tests verbal encouragement was given to each subject.

**Fig. 1:**
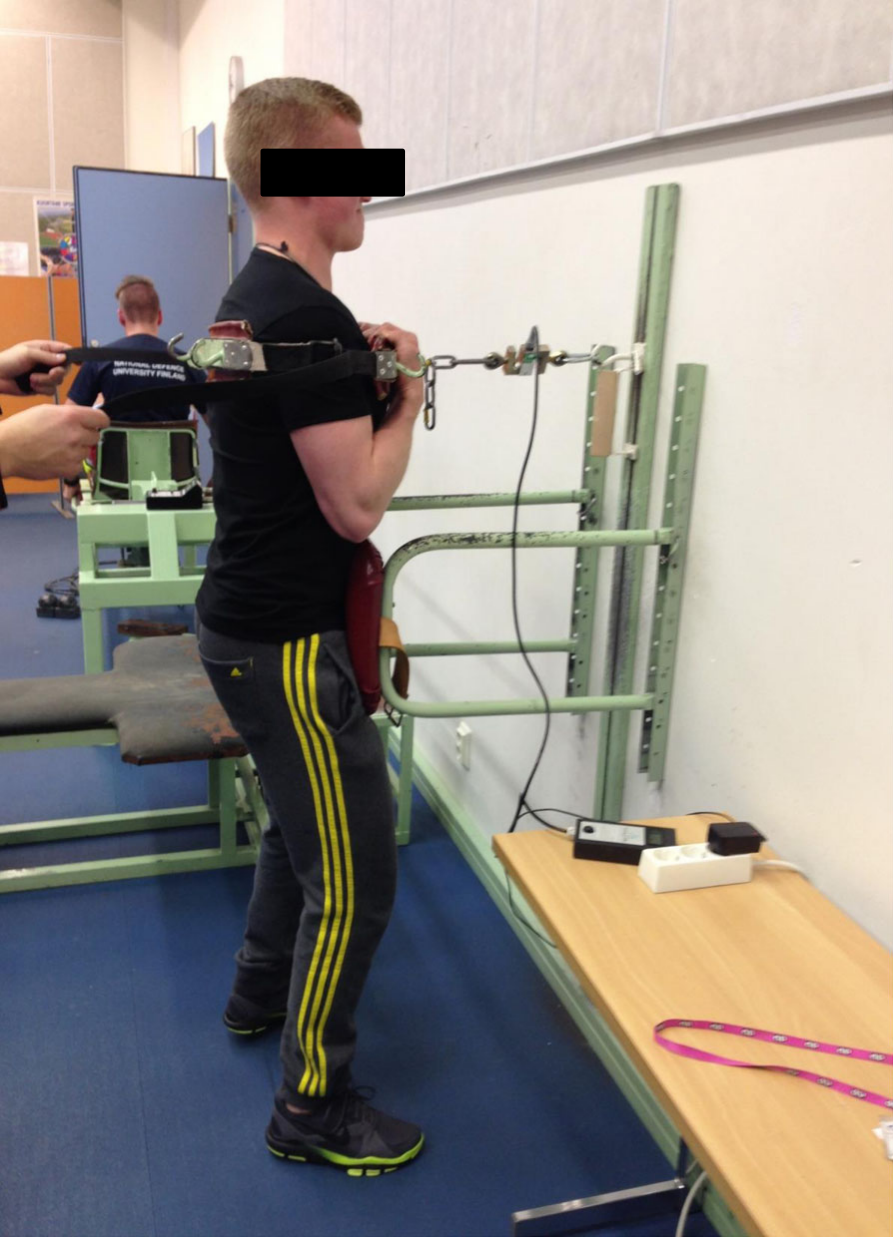
Maximal isometric trunk extension

Maximal isometric trunk flexion and extension were performed in the standing position. The extension test is shown on the Fig. 1, while the flexion test is done in the same aperture standing the opposite way (face away from the wall). The measurement was recorded by an isometric strain-gauge dynamometer [25]. The hips were fixed at the level of the anterior superior iliac spine. The strap was tightened around the shoulders just below the armpit and horizontally connected to the dynamometer (Digitest LTD, Oulu, Finland) by a steel chain. A minimum of two trials was performed for each subject and the best result was selected for further analysis. The duration of maximal pull against the strap was held for 3–5 s and performed twice with 30–60 s rest between the sets.

**Fig. 2:**
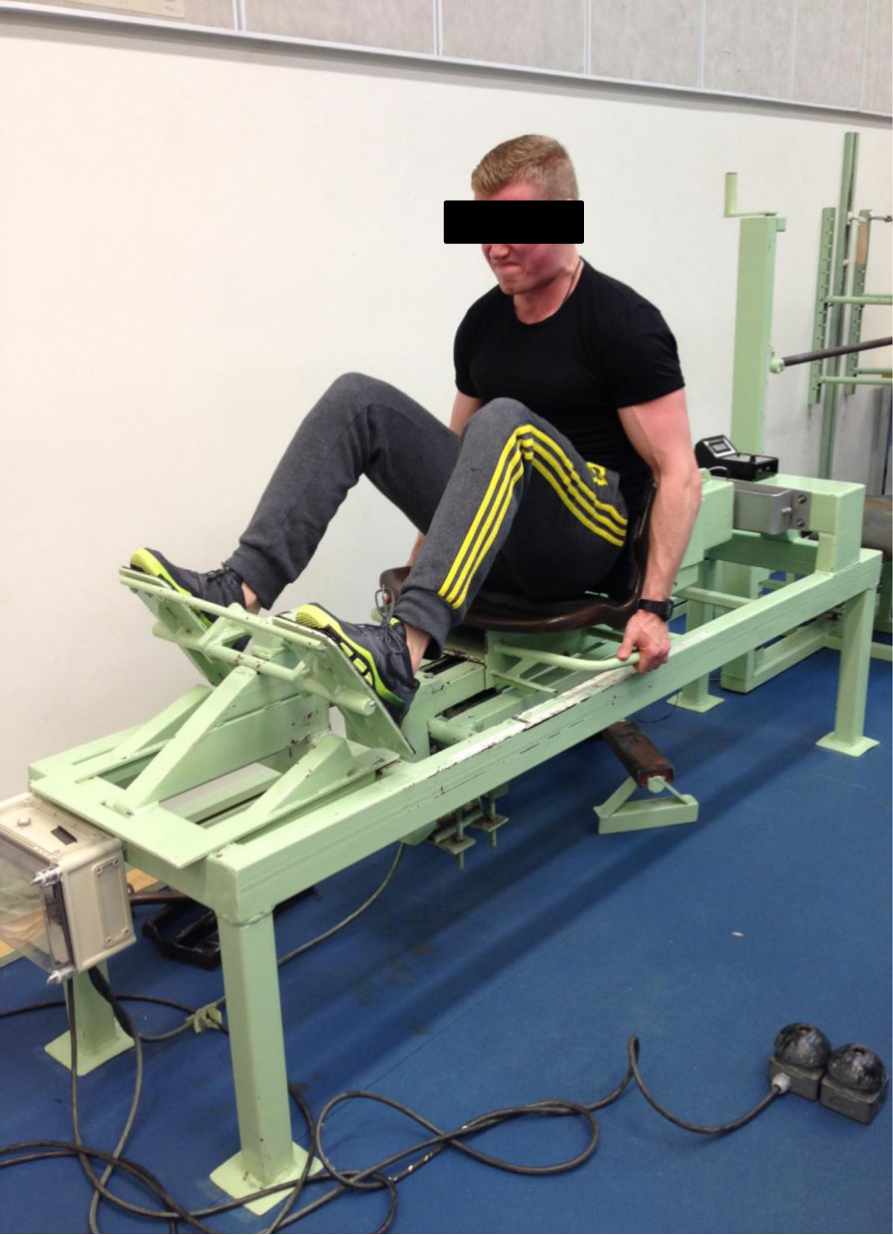
Maximal isometric bilateral leg extension

Maximal isometric bilateral leg extension force (Fig. 2) was measured on an electromechanical dynamometer. The subject was positioned sitting on the bench with their back firmly fixed into the backrest and hands on the handles. The subjects placed their feet on the resistance stand at the base of the sledge. The knee angle was set to 90 degrees using a goniometer. The maximal push towards the leg stand was held for 3–5 s and performed twice with 30–60 s rest between the sets. The measurement was recorded by an isometric strain-gauge dynamometer. A minimum of two trials were performed for each subject and the best result was selected for further analysis. This method is well documented and used in many previous studies [26, 27]. The reproducibility of measurements of maximal isometric muscle force is high (*r* = 0.98, C.V. =4.1%) [28]. Finally, overall maximal muscle strength in the present study refers to the results of these three measurements (leg extension and trunk flexion and extension).

### Physical activity, pain and disability questionnaire

Each participant was questioned about their history of sport and exercise participation and LBP symptoms during the follow-up period. The structured questionnaire included questions of musculoskeletal disorders during the last year and for the whole follow-up period. There was a section for each (lumbar, thoracic and cervical) region which all was pictured in a questionnaire to validate the localized symptoms. If the pain was ongoing or the subject had experienced pain during the last seven days prior to filling in the questionnaire, the value of the visual analogic scale (VAS) was also questioned. Questions related to physical activity level were: “How many days had the subject had been physically active (exhaustive exercise which includes both increased ventilation and sweating for at least 30 min) during the last week as well as during the last days?” The annual activity level was asked separately for aerobic exercises (i.e. running, cross country skiing, etc.), muscular strength (i.e. cross fit, resistance training and martial arts, etc.) and racket (i.e. tennis) and ball games (i.e. soccer, basketball, ice-hockey, etc.). The subject was asked to name the sports he had participated in.

### Statistical analysis

Means with standard deviations (± SD) are given as descriptive statistics. Shaprio-Wilk’s test was used to test the assumption of normality. Relationships between muscle CSA, composition and strength test results were evaluated using Pearson’s correlation coefficient (r). A one-way repeated measured analysis of variance (ANOVA) was conducted to evaluate the null hypothesis that there is no change in subjects’ CSA during the 5-year follow-up. Further analysis to explore the predictive value of the CSA measurements were performed, and the subjects were divided into LBP and non-LBP groups. The student’s t-test was used for comparison between the groups. The level of significance was set at 0.05. All analyses were conducted using SPSS Statistics for Windows V.21.0 software.

## Results

The mean (±SD) CSA of the paraspinal muscles among the study group was 31.0 (3.8) cm^2^ at the L3–4 and 28.6 (3.8) cm^2^ at L4–5 levels. The mean CSA of the psoas muscle was 25.7 (3.4) cm^2^ and 21.3 (3.2) cm^2^, respectively. All the subjects were ranked in category 2 (normal) in the 3-point (0–2) visual scale measuring muscle composition. The descriptive values of the maximal isometric strength test results are presented in Table 1. The mean self-reported sport participation was 3.2 times per week for overall sports participation and 1.9 for strength training, respectively.

The follow-up comparisons indicated that there was a statistically significant (*p* < 0.01) increase in CSA of the paraspinal muscles over the 5-year follow-up period. The mean CSA of the paraspinal muscles (left and right side combined) increased by 8 and 7% at the L3–4 and L4–5 levels, respectively, during the 5-year follow-up. However, the increase in CSA of the psoas muscles (2% at L3–4 and 3% at L4–5) was statistically not significant. CSAs in all measurement points are described in Table 2.

**Table 2:** Longitudinal changes of CSA (cm^2^) of the paraspinal and psoas muscles (mean ± SD)

	Baseline	Follow-up	*P* ^*a*^	95% CI
PS 3–4 (R)	31.3 (4.0)	33.7 (4.3)	< 0.01**	1.6 to 3.2
PS 3–4 (L)	30.7 (3.3)	33.2 (3.5)	< 0.01**	1.6 to 3.4
PS 4–5 (R)	28. 7 (3.8)	30.1 (4.2)	< 0.01**	0.7 to 2.2
PS 4–5 (L)	28.5 (4.3)	30.9 (4.7)	< 0.01**	1.5 to 3.3
Psoas 3–4 (R)	16.9 (3.4)	17.4 (3.3)	0.02*	0.1 to 0.9
Psoas 3–4 (L)	17.4 (3.6)	17.7 (3.5)	0.27	0.3 to 0.9
Psoas 4–5 (R)	21.0 (3.2)	21.8 (3.9)	0.07	0.1 to 1.6
Psoas 4–5 (L)	21.5 (3.2)	22.2 (3.4)	0.05	0.1 to 1.3

*CSA* Cross-sectional area, *PS* Paraspinal muscles, *R* right, *L* left; ^a^ANOVA

for repeated measures, (Wilks’ Lambda) was used to obtain *P values;* *p < 0.05; **p < 0.01

The mean (±SD) combined CSA of psoas was 15.9 (3.0) cm^2^ at L 3–4 and 20.1 (3.0) at L4–5, respectively among the pilots who not experienced LBP. The CSAs of psoas among the symptomless counterparts were 17.7 (3.5) cm^2^ at the L 3–4 and 21.8 (3.2) at L4–5 level, respectively. The difference was statistically not significant in either at L3–4 (*p* = 0.21) or at L4–5 (p = 0.21). There was also no statistically significant difference in CSA of the paraspinal muscle. At the L3–4 level it was 31.2 cm^2^ (4.0) among pilots who had experienced LBP and 30.9 cm^2^ (3.7) among the symptomless counterparts. The results at the level of L4–5 were 29.1 (5.6) and 28.3 (2.9), cm^2^, respectively. The difference was statistically not significant in either at L3–4 (p 0.89) or at L4–5 (p 0.64).

There was a statistically significant correlation with the leg extension test results and the combined (left and right side) CSA of the psoas (*r* = 0.60, *p* < 0.01) and paraspinal muscles (*r* = 0.60, *p* < 0.01) at the L3–4 level. Table 3 shows that there were also statistically significant correlations between the trunk flexion and extension test results and side to side paraspinal muscle CSA at the L3–4 and L4–5 levels and CSA of the psoas muscles at the L4–5 level. The correlation coefficients at each CSA measuring point are presented in Table 4.

**Table 3:** Correlations coefficients (r) between combined (left and right side) CSA measurement and strength test

	Leg Extension		Trunk Flexion		Trunk Extension	
	r	*P*	r	*P*	r	*P*
PS 3–4	0.60**	<.01	0.50**	<.01	0.50**	<.01
PS 4–5	0.23	.26	0.44*	.03	0.43*	.03
Pso 3–4	0.60**	<.01	0.38	.06	0.36	.07
Pso 4–5	0.54**	<.01	0.48*	.01	0.45*	.02

*PS* Paraspinal Muscles, *Pso* Psoas Muscles, *CC* Correlation Coefficient (Spearman); *p < 0.05; **p < 0.01

**Table 4:** Correlations coefficients (r) between side to side CSA measurement and strength test results

	Leg Extension		Trunk Flexion		Trunk Extension	
	r	*P*	r	*P*	r	*P*
PS 3–4 (R)	0.59**	< 0.01	0.47*	0.02	0.52**	0.01
PS 3–4 (L)	0.60**	< 0.01	0.53**	0.01	0.46*	0.02
PS 4–5 (R)	0.29	0.15	0.36	0.08	0.42*	0.03
PS 4–5 (L)	0.15	0.47	0.47*	0.02	0.40*	0.04
Pso 3–4 (R)	0.68**	< 0.01	0.39*	0.04	0.39	0.05
Pso 3–4 (L)	0.51**	0.01	0.35	0.08	032	0.11
Pso 4–5 (R)	0.60**	< 0.01	0.48*	0.01	0.48*	0.01
Pso 4–5 (L)	0.47*	0.02	0.47*	0.02	0.40*	0.04

*PS* Paraspinal Muscles, *Pso* Psoas Muscles, *CC* Correlation Coefficient (Spearman); *p < 0.05; **p < 0.01

In further analysis, CSA between pilots who had experienced LBP and their symptomless pilots during the follow-up revealed that there was no statistically significant difference between the LBP group (*n* = 8) and symptomless (*n* = 18) group. Furthermore, there was no statistical difference between the side-to-side asymmetry between the pilots who had experienced LBP and the pilots who had been symptomless.

## Discussion

The present study demonstrated that the muscle CSA increased in all measured segments (L3 - L4 and L4 - L5) both in the psoas and paraspinal muscles during the 5-year follow-up. However, the increase in CSA was statistically significant in both sides of the paraspinal muscles in L3 - L4 and L4 - L5 but only at the right side of the psoas muscle at the L3–4 level. At the baseline, it was further found out that the maximal leg extension force correlated with the psoas and paraspinal muscles’ CSA, with the exception of psoas CSA at the L3–4 level. In addition, both maximal trunk extension and flexion forces correlated with paraspinal muscles CSA in L3 - L4 and L4 - L5 and psoas CSA in L4–5 at the baseline.

Increased muscle CSA is generally expected following a resistance training intervention of sufficient duration and workload [29, 30]. It has been suggested that maximal trunk extension and flexion forces correlate with CSA of the paraspinal and psoas muscles [17]. Furthermore, Gibbons et al. [31] found out in their twin study that an intensive bodybuilder had 27% greater CSA of the erector spinae muscle than that of his twin. However, it is not possible to conclude if the muscular strength had increased along with the increase of muscle CSA among the subjects of the present study because only the baseline strength test results were available. According to the results of the health questionnaire, our subjects were physically active individuals. The average amount of sports participation was more than three times per week and 15 out of 26 subjects reported doing strength training at least twice a week regularly throughout the year. Therefore, we suggest that a part of the increased CSA could be a result of regular resistance training. The anti G straining maneuver (AGSM) executed during the high-performance flying includes isometric muscle contractions which could also theoretically lead to muscle mass increase. Although, the proper AGSM is done mainly by contracting thigh, buttock and abdominal muscles, the high performance flying itself may also cause the part of the increase of CSA reported in the present study.

An increased amount of fat is normally the first change in muscles of the lower back due to inactivity. In the present study, the composition of the paraspinal or psoas muscles did not change over the follow-up period, although body weight increased. This finding was in contrast to the findings of the longitudinal (15-yr follow-up) study of Fortin et al. [20] which suggests that age is significantly associated with composition of the paraspinal muscles. Nonetheless, the finding of the present study was expected due to a relatively short follow-up period and the young age of the subjects. For example, the follow-up period of the longitudinal study of Fortin et al. [20] was three times longer and the mean age of subjects were older (47 yrs. vs. 21 yrs.) than in the present study.

Previous studies investigating CSA of the paraspinal muscles have reported a caudal increase in CSA of the multifidus and decrease of the erector spinae muscles [13]. In the present study, CSA of the multifidus and erector spinae muscles were measured together as one muscle mass (paraspinal muscles). Therefore, it is not possible to define if there was caudal increase in the multifidus muscle only. In accordance with previous literature investigating the multifidus and erector spinae muscles together, we also found out that CSA was larger at L3-L4 than at L-4 - L5 [22]. The results of this study showed only a little side to side asymmetry of CSA between the measured muscles. The mean CSA measurements of the paraspinal muscles were slightly larger on the right side as compared to the left side in the baseline measurements. The difference between the mean CSA of the paraspinal muscles was 0.60 cm^2^ (31.29–30.69 cm^2^) at L3 - L4 and 0.18 cm^2^ (28.67–28.49 cm^2^) at L4 - L5, and the difference was not statistically significant.

In this study, a statistically significant correlation was found between isometric strength test results and CSA of the measured muscles at the baseline. It indicates that muscles with larger CSA are capable of producing more power in isometric strength tests. The trunk flexion and extension test results had significant correlation in both levels (L3–4 and L4–5) of the CSA measurements of the paraspinal muscles. Furthermore, both test results correlated with psoas CSA measurement at the L4–5 level. These findings support previous research [17, 18] where CSA of the paraspinal and psoas muscles have been associated with isokinetic and isometric strength test results. Nonetheless, there are conflicting results. Parkkola et al. [16] could not find association between maximum isometric extension strength and CSA of the lumbar muscles among medical students aged between 21 and 27 years. This contradictory finding could be explained with differences in sex and physical training. Furthermore, the subjects in the current study were active males, whereas Parkkola et al. [16] studied sedentary women.

The leg extension test results showed a significant correlation between CSA of the psoas muscle at levels L3–4 and L4–5. Furthermore, the leg extension test correlated with the paraspinal CSA measurement at L3–4 level. The investigators were not able to find research discussing directly the association between the strength of lower limb muscles and CSA and composition of the lumbar paraspinal or psoas muscles. Therefore, this finding can be considered as novel. The explanation to why the CSA of the psoas muscles correlated with the maximal force production of the leg extensors is not clear. It has been reported that the psoas muscles’ CSA as well as lower limb (quadriceps and adductor) muscles’ CSA correlates with sprint velocity [32]. Furthermore, it has been found that high intensity training improves not only lower limb but also trunk muscle hypertrophy [33]. Therefore, it is possible to speculate that those subjects who are capable of producing greater force with the lower limb extensors (i.e. rectus femoris and gluteus) may also have larger psoas muscles.

CSA or muscle composition of the studied muscles did not have predictive role on LBP in the 5-year follow-up and supports previous research [14, 20].There are also conflicting results suggesting that muscle composition [6] and CSA [10] of the multifidus muscle is associated with LBP and self-reported disability [34]. Thus, the relationships between muscle composition and CSA and LBP have been found with subjects with a mean age of between 37 and 40 years [6, 10]. When discussing the predictive role of muscle CSA and composition, the most important limitation with these previous studies is the cross-sectional design. The direction, whether the abnormal muscle is the cause of LBP or vice versa, should be investigated in longitudinal studies. Moreover, because the association between muscle strength and LBP was not found in the present 5-year follow-up, it is suggested that longer follow-up studies should be done to investigate the relationship between LBP and muscle strength. However, unless there is no other evidence, it is also justified to say that muscle CSA may not be important in dealing with LBP or risk for pain.

The use of reliable/valid methods in this investigation enhances the quality of the study. The reliability of muscle CSA measurements performed with an MRI is well supported [23, 24]. In addition to high reliability of muscle measurements with MRI scanning, also physical fitness measurements used in this study have been used in several previous studies [26, 27] and their reproducibility is high [28]. A limitation to this study is that there were only strength measurements during baseline.

The 5-year follow-up period of the young fighter pilots may be too short when discussing the relationship between CSA of muscles and LBP and the flight related pain in particular. The subjects only had a few years of the +Gz exposure (flying with fighter jets), which may be the reason that only eight of 26 subjects reported of any kind of LBP episode in the follow-up. Conversely, Rintala et al. [3] found that 9 out of 10 FINAF pilots have experienced musculoskeletal disorders already during their fighter training. The reason for conflicting results might be due to different kind of questionnaires and the subjective nature of these investigations. Furthermore, musculoskeletal disorders studied in the study of Rintala et al. [3] included disorders in both cervical and lumbar areas.

## Conclusions

In summary, this was the first study to evaluate lumbar paraspinal muscle composition and CSA among fighter pilots. The present 5-year follow up study suggests that over the first five years of flight service, paraspinal muscles’ CSA increases and associates well with the baseline strength test results among the FINAF fighter pilots. Therefore, it could be concluded that in spite of the fact the strength levels of FINAF fighter pilots might increase during the first five years of their career, no association between future LBP and MRI findings of paraspinal or psoas muscles’ CSA was observed. Nevertheless, the LBP occurrence was low among the study population, and therefore, we recommend future studies to investigate this association with longer follow-up periods.

## Abbreviations

CSA: Cross sectional area
FINAF: Finnish Air Force
LBP: Low back pain
MRI: Magnetic resonance imaging
VAS: Visual analogic scale
